# A Cross-Sectional Evaluation of Cigarette Smoking in the Brazilian Youth Population

**DOI:** 10.3389/fpubh.2021.614592

**Published:** 2021-11-04

**Authors:** Emerson Silveira Brito, Marina Bessel, Thayane Dornelles, Flávia Moreno, Gerson Pereira, Eliana Márcia Da Ros Wendland

**Affiliations:** ^1^Hospital Moinhos de Vento, Porto Alegre, Brazil; ^2^Programa de Pós-Graduação em Ciências da Saúde, Federal University of Health Science of Porto Alegre, Porto Alegre, Brazil; ^3^Department of Chronic Diseases Conditions and Sexually Transmitted Diseases, Ministry of Health, Brasilia, Brazil

**Keywords:** smoking, youth population, smoking—epidemiology, cigarette addicted students, cigarette smoking

## Abstract

**Introduction:** The transition from adolescence to adulthood involves a variety of physical, behavioral, and social transformations, often including tobacco use. Because understanding smoking at this stage is important for tobacco control, we aimed to analyze the prevalence of cigarette smoking in the Brazilian youth population.

**Methods:** This study included participants aged 16–25 years from all Brazilian capitals. A standardized questionnaire was administered by trained healthcare professionals to collect information about sociodemographic status, sexual behavior, and tobacco use. The samples from each capital were weighted by age range and sex.

**Results:** Of the 8,581 participants, 15.1% were current smokers, and 20.0% were former smokers; the average age at first tobacco use was 15.5 years. The prevalence of smoking in men was higher than that in women (20.1 vs. 10.3%, *p* < 0.01). Education level was associated with current smoking and former smoking. Participants with an elementary education level had a higher smoking prevalence (PR: 5.84, 95%, CI: 4.29–7.95) than those with a secondary education (PR: 2.19, 95% CI: 1.63–2.93) and those with higher education. Those without current partners (PR: 1.27, 95% CI: 1.03–1.56) also had a higher prevalence of smoking than those with partners, and participants who had a previous same-sex sexual experience smoked more (PR: 2.29, 95% CI: 1.78–2.96) than those who did not. In addition, regular use of alcohol was associated with higher prevalence of cigarette smoking (PR: 5.65, 95% CI: 4.03–7.90) than a lack of alcohol consumption. Skin color and socioeconomic class did not exhibit significant relationships with tobacco use patterns.

**Conclusions:** Smoking was associated with education level regardless of social class, and some specific behaviors associated with a same-sex sexual experience were associated with a higher prevalence of smoking. These findings are important for formulating policies and directing actions to combat and prevent smoking among young populations.

## Introduction

Cigarette smoking, which is the most common form of tobacco use (1), has negative impacts on health worldwide due to its association with chronic non-communicable diseases and cancer (2–5). It is estimated that 1.3 billion people are active smokers worldwide (6). Every year, five million deaths due to smoking-related diseases occur in low- and middle-income countries (7). In Brazil, it is estimated that 24 million people smoke and that 16.9% of the population has used tobacco at least once in their lives (4, 8). In the last 10 years, the prevalence of smoking has decreased in all groups in Brazil except among individuals aged 18–24 years (9, 10).

The transition from adolescence to adulthood involves a variety of physical, behavioral, and social transformations, including tobacco use (11, 12). Licit drugs, such as tobacco, have high rates of use across the globe and are usually the second substance that adolescents and adults try due to curiosity, a wish to be accepted in a group, or a desire to rebel (8, 9, 13–15). Individual factors such as sex, age, education level, wellness, and the consumption of other substances, such as alcohol and illicit drugs, are related to tobacco use (16, 17). Other factors associated with smoking have also been identified, such as early sexual initiation, suicidal ideation, the absence of ties to religion, exposure to smoking products via parents, difficulty sleeping, absence of close friends, and the experience of family violence (18–20). Additionally, family, culture, and geographic location affect tobacco use (21, 22).

Smoking at an early age makes it difficult to stop smoking in adulthood (23, 24). However, for those who quit using tobacco before the age of 30 years, many of the negative consequences of tobacco use are reduced (14). While smoking among young people is a problem in all social groups, the lack of current data from various populations also remains a major problem (25, 26). Cigarette use among young people is a health-related behavior that can persist into adulthood (27–29). Therefore, understanding smoking behavior during the transition to adulthood is important for tobacco control at this stage. Thus, this study aimed to analyze cigarette smoking and its associated factors among the youth population of Brazil.

## Materials and Methods

This was a multicenter, cross-sectional study that assessed the prevalence of human papillomavirus (HPV) and other behaviors, including tobacco use (POP-Brazil study), in the capitals of all 26 Brazilian states plus the Federal District (30). The study, which included participants aged 16–25 years from 119 primary health units, was conducted by trained health professionals and took place between September 2016 and December 2017. The research methods consisted of the application of a standardized questionnaire based on validated instruments that appraised sociodemographic variables, smoking, alcohol consumption, other drug consumption status, and sexual and reproductive health data.

All participants in the target age range who lived in the areas of the primary health units were invited to participate. Although we used a convenience sampling method to select participants, different approaches were used to minimize selection bias. We invited participants from a list of all patients living in the primary healthcare area, those who responded to invitations in schools, those who responded to invitations in communities made by community health agents, and individuals who sought care or were accompanying people seeking care at the primary health care units. All professionals included in the data collection process were personally trained by the study research group. Training topics included recruitment techniques, requests for consent, and interview methods. Data collection was conducted by trained professionals who asked the questions and entered the responses into the data system that was designed specifically for the study. The interview lasted approximately 40 min. A pilot study was conducted from September 2016 to December 2016 in two Brazilian capitals, including 217 participants, to adjust the instruments and logistics.

All respondents participated in a standardized interview that collected data on their age, sex, self-declared race/skin color, household characteristics, relationship status (without a partner: single or with a partner: dating, married, living with a partner), education level (low education: complete or incomplete elementary school, middle education: complete or incomplete secondary school, and high education: University level or higher), number of children (only for women), occupational status, household income in the previous month, and same-sex experience [“Have you had a same-sex sexual experience in the past 12 months?” (31, 32)]. Participants who did not report having had sexual intercourse in the last 12 months were excluded from the analysis of this variable. The socioeconomic class was defined using the Brazilian Criteria of Economic Classifications, which is based on asset ownership (33). Smoking was defined as regular cigarette use in the past year (daily current smoking or non-daily current smoking vs. no smoking). Participants were also asked about their current and past smoking habits (“Do you currently smoke cigarettes?”, “Have you smoked cigarettes in the past?”, “How many cigarettes do you smoke per day and week?”, “How old were you when you started smoking?”). Self-reported alcohol intake was evaluated (regular use: daily, more than once per week, 2–3 times per month; occasional use: a few times per year or once per year; and no alcohol consumption).

The study was conducted according to the regulations of the Brazilian legislation (CNS 466/2012), and the protocol was approved by the Research Ethics Committee of the Moinhos de Vento Hospital (number 1607032) and all coparticipating institutions. All participants provided written consent after being informed of the study procedures.

Regarding the calculation of the sample size, differences of 5% were considered in the regions where HPV was relevant and exhibited a power of 80 and an alpha error of 5% for women, using a 30% estimate for the prevalence of HPV in Brazil. Thus, 7,935 individuals were required, i.e., 1,587 participants from each of the five regions of Brazil, to maximize diversity in the less populated areas and standardization during the analyses (30). Of the eligible participants, 99.08% agreed to participate in the study, and the percentage response rate was 99.9%, resulting in 8,581 subjects.

The selection of the variables was based on the literature that defines socioeconomic criteria, sociodemographic characteristics, and smoking indicators (31–33). Descriptive statistics were calculated, and Pearson's chi-square test or Fisher's exact test was used to compare the prevalences of categorical variables. Furthermore, Poisson multivariate regression with robust variance was performed based on a hierarchical model to compute the associations between different variables and smoking. The multivariate models were adjusted for sociodemographic variables (sex, age, skin color, region, socioeconomic status, and education level), and the behavioral variables were added to model 2 (partner presence, sexual experience, and alcohol use). To adjust the distribution of the sample to the study population, we weighted the measures based on the size of the population in each capital and sex. Finally, a statistical analysis was performed using SAS software V.9.4 (Statistical Analysis System, SAS Institute), and a statistical significance level of 5% was adopted. The answer “I do not know” was considered a missing value. The raw data supporting the conclusions of this study will be made available by the authors without undue reservation.

## Results

**Figure 1:**
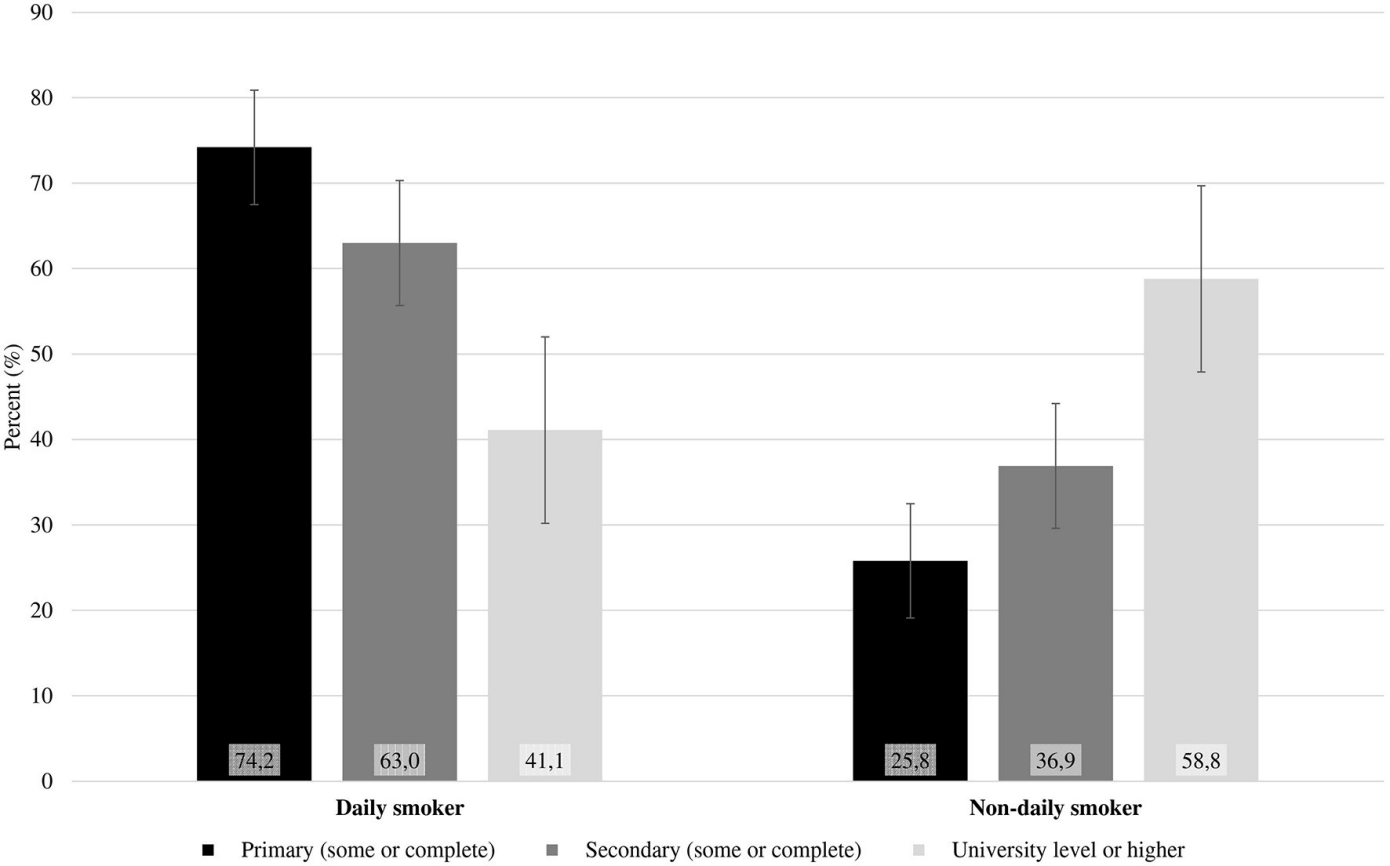
Education level in daily and non-daily current smokers among the youth population of Brazil, 2016–2017.

In all, 8,581 participants (50.8% female) were surveyed. Among them, 15.1% were current smokers, and 20.0% were former smokers. The mean age at first tobacco use was 15.5 (95% CI: 15.20–15.74) years. The majority of smokers smoked fewer than 20 cigarettes per day (80.9%). Among the current smokers, 65.2% smoked cigarettes daily, and the mean number of cigarettes smoked was 10.3 per day (95% CI: 9.0–11.5), which was higher than 7.4 per week smoked by non-daily current smokers (95% CI: 5.6–9.2). Sex differences were observed in socioeconomic status, education level, having a partner, same-sex sexual experience, and alcohol use (*p* < 0.01) (Table 1). The prevalence of smoking was higher in the southern region of Brazil and higher among people with lower education levels, people without partners, women without children, people with a same-sex sexual experiences, and people who consumed alcohol regularly (Table 2). There were no statistically significant differences in self-declared skin color/race or socioeconomic status. While there was a decreased frequency of daily smoking according to education level (74.2% at the primary level to 41.1% at the University level), nondaily current smokers exhibited the opposite pattern (25.8% at the primary level to 58.8% at the University level) (*p* < 0.01) (Figure 1).

**Table 1:** Sociodemographic characteristics of the participants aged 16–25 years in Brazil according to sex, 2016–2017.

**Variables**	**Overall (%)**	**Female (*n* = 6,366) *n* (%)**	**Male (*n* = 2,215) *n* (%)**	***p*-value^a^**
**Age, years (21.4)**				
≤ 18	1,086 (12.6)	839 (13.2)	247 (11.1)	0.9
> 18	7,495 (87.4)	5,527 (86.8)	1,968 (88.9)	
**Race/skin color**				0.8
White	2,086 (23.9)	1,556 (23.3)	530 (24.5)	
Black	1,359 (16.8)	993 (16.5)	366 (17.1)	
Pardo/Brown	4,882 (56.8)	3,630 (57.5)	1,252 (55.9)	
Others	203 (2.5)	157 (2.6)	46 (2.4)	
**Region**				0.9
Midwest	1,827 (12.4)	1,084 (12.0)	743 (12.7)	
Northeast	2,164 (27.3)	1,653 (27.1)	511 (26.8)	
North	1,729 (12.3)	1,348 (12.3)	381 (12.3)	
Southeast	1,555 (40.4)	1,220 (40.4)	335 (40.5)	
South	1,306 (7.6)	1061 (7.4)	245 (7.7)	
**Socioeconomic status**				<0.01
Low	2,373 (26.0)	1,997 (32.1)	376 (19.7)	
Middle	4,578 (55.7)	3,404 (54.5)	1,174 (57.0)	
High	1,630 (12.8)	965 (13.3)	665 (23.2)	
**Education level**				0.07
Elementary	1,819 (23.6)	1,429 (24.9)	390 (22.2)	
Secondary	4,784 (55.1)	3,541 (55.4)	1,243 (54.7)	
University	1,976 (21.3)	1,394 (19.7)	582 (23.0)	
**Partner**				<0.01
Without a partner	6,510 (75.8)	5,058 (80.4)	1,452 (71.0)	
With a partner	2,069 (24.2)	1,306 (19.6)	763 (29.9)	
**Number of children^b^**				-
0	258 (9.7)	258 (9.7)	-	
1	1,945(65.7)	1,945 (65.7)	-	
≥ 2	774 (24.6)	774 (24.6)	-	
**Sexual experience**				<0.01
Heterosexual	7,055 (92.6)	5,540 (96.4)	1,515 (88.0)	
Same-sex	416 (7.3)	238 (3.6)	178 (12.0)	
**Alcohol use**				<0.01
Regular consumption	3,153 (40.5)	2,098 (32.5)	1,055 (48.7)	
Occasional consumption	2,406 (26.0)	1,839 (27.7)	567 (24.2)	
No use	3,013 (33.5)	2,423 (39.8)	590 (27.0)	

a *Chi-square test*.

b *Only for females*.

**Table 2:** Smoking status according to sociodemographic and behavioral characteristics of the participants aged 16–25 years in Brazil, 2016–2017.

	**Smoking status**			
**Variables**	**Non–smoker (*N* = 5,385) % (95% CI)**	**Current smoker (*N* = 1,119) % (95% CI)**	**Former smoker (*N* = 1,626) % (95% CI)**	***p*–value^a^**
**Sex**				<0.01
Male	56.8 (53.4–60.1)	20.1 (17.4–22.9)	23.1 (20.3–25.9)	
Female	72.8 (71.1–74.4)	10.3 (9.1–11.4)	16.9 (15.6–18.3)	
**Age, years (mean** **=** **21.4)**				0.26
≤ 18	60.8 (55.3–63.6)	17.3 (12.6–22.3)	21.9 (17.5–26.4)	
> 18	65.6 (63.6–67.6)	14.7 (14.7–13.2)	19.6 (17.9–21.3)	
**Race/skin color**				0.81
White	65.0 (61.1–68.9)	14.2 (11.3–17.1)	20.8 (17.5–24.1)	
Black	63.6 (58.9–68.3)	14.8 (11.1–18.5)	21.6 (17.6–25.6)	
Pardo/Brown	65.5 (62.9–67.9)	15.7 (13.6–17.7)	18.9 (16.9–20.9)	
Others	59.9 (46.1–13.7)	15.2 (4.7–25.8)	24.8 (12.4–37.3)	
**Region**				0.02
Midwest	64.1 (60.3–68.0)	15.7 (12.8–18.7)	20.1 (16.8–23.3)	
Northeast	64.9 (61.9–67.9)	13.7 (11.3–16.0)	21.4 (18.9–24.0)	
North	66.0 (62.3–69.8)	11.9 (9.1–14.7)	22.1 (18.8–25.4)	
Southeast	66.3 (62.5–70.1)	15.9 (12.9–18.9)	17.8 (14.7–20.8)	
South	57.0 (52.7–61.4)	20.1 (16.5–23.7)	22.8 (19.1–26.5)	
**Socioeconomic status**				0.10
Low	61.0 (57.5–64.5)	17.9 (15.1–20.8)	21.0 (18.1–23.9)	
Middle	66.8 (64.2–69.3)	14.1 (12.1–16.1)	19.1 (17.1–21.2)	
High	64.8 (60.3–69.3)	14.2 (10.9–17.4)	21.0 (18.1–23.9)	
**Education level**				<0.01
Elementary	49.2 (45.1–53.3)	25.7 (21.9–29.5)	25.1 (21.6–28.5)	
Secondary	67.9 (65.3–70.4)	13.6 (11.6–15.5)	18.6 (16.5–20.6)	
University	74.6 (71.1–78.1)	7.4 (5.8–9.1)	17.9 (14.6–21.2)	
**Partner**				0.03
With a partner	66.2 (64.0–68.3)	14.1 (12.5–15.8)	19.7 (17.9–21.5)	
Without a partner	61.0 (56.9–64.9)	18.3 (15.0–21.5)	20.8 (17.5–24.1)	
**Number of children^b^**				<0.01
0	51.1 (41.9–60.4)	22.2 (14.0–33.3)	26.7 (18.4–31.5)	
1	71.4 (68.7–70.0)	11.4 (9.5–13.3)	17.2 (15.1–19.4)	
≥ 2	64.9 (53.9–75.8)	16.9 (7.3–26.5)	18.2 (11.2–25.3)	
**Sexual experience**				<0.01
Heterosexual	68.8 (66.8–70.8)	12.5 (11.0–13.9)	18.7 (17.1–20.4)	
Same–sex	42.3 (34.7–49.9)	30.5 (22.7–38.3)	27.1 (20.6–33.6)	
**Alcohol use**				<0.01
Regular consumption	44.1 (40.9–47.3)	28.1 (25.1–31.1)	27.7 (24.8–30.6)	
Occasional consumption	70.4 (67.2–73.6)	9.4 (7.1–11.6)	20.2 (17.5–22.9)	
No use	85.8 (83.4–88.1)	3.8 (2.7–5.0)	10.4 (8.3–12.5)	

a *Chi–square test*.

b *Only in females*.

A multivariate analysis was performed to verify the variables associated with smoking status, and the estimates were adjusted for all variables presented in the table. The results showed that the frequency of current smoking was lower for females (0.46; 95% CI: 0.39–0.54), higher for those who had had a same-sex sexual experience (2.29; 95% CI: 1.78–2.96), and higher for those who consumed alcohol in all models for both current (5.65; 95% CI: 4.03–7.90) and former smokers (3.83; 95% CI: 2.88–5.09) (Table 3). Moreover, current smoking was inversely associated with education level (elementary 5.84; 95% CI: 4.29–7.95; secondary 2.19; 95% CI: 1.63–2.93). Those who did not have a partner were relatively more likely to be current smokers (1.27; 95% CI: 1.03–1.56) but not former smokers (1.03; 95% CI: 0.85–1.24). In the fully adjusted analysis, the northern region exhibited a higher prevalence of former smoking than did the northeastern, midwestern, and southeastern regions. In general, in the multivariate model, when the behavioral variable was included in the model (fully adjusted model), there was a decrease in the magnitude or even the disappearance of the significance of the estimates, except for education level, which showed an increase in the magnitude of the effect (4.07; 95% CI: 3.07–5.41 to 5.84; 95% CI: 4.29–7.95 at the elementary level, and 1.98; 95% CI: 1.52–2.59 to 2.19; 95% CI: 1.63–2.93 at the secondary level). Finally, it was determined that age, skin color, and social class were not associated with either current or former smoking.

**Table 3:** Factors associated with smoking in a multivariate analysis of Brazilian population, 2016–2017.

	**Current smoker**		**Former smoker**	
**Variables**	**Model 1**	**Model 2**	**Model 1**	**Model 2**
	**PR (95% CI)**	**PR (95% CI)**	**PR (95% CI)**	**PR (95% CI)**
**Sex**				
Male	1	1	1	1
Female	0.46 (0.39–0.54)	0.55 (0.46–0.66)	0.64 (0.55–0.74)	0.71 (0.61; 0.84)
**Age, years**				
<18	1	1	1	1
≥18	0.98 (0.75–1.27)	0.89 (0.71–1.12)	0.94 (0.75–1.16)	0.81 (0.66; 1.00)
**Race/skin color**				
White	1	1	1	1
Black	1.03 (0.76–1.41)	1.01 (0.75–1.36)	1.04 (0.81–1.32)	1.01 (0.79–1.29)
Brown	1.13 (0.87–1.45)	1.04 (0.82–1.33)	0.93 (0.77–1.14)	0.99 (0.81–1.23)
Other	1.04 (0.56–1.91)	0.96 (0.44–2.09)	1.21 (0.73–2.02)	1.27 (0.83–1.94)
**Region**				
North	1	1	1	1
Midwest	1.47 (1.11–1.94)	0.96 (0.71–1.29)	0.96 (0.77–1.19)	0.77 (0.61–0.96)
Northeast	1.12 (0.87–1.46)	0.77 (0.59–0.99)	0.95 (0.79–1.15)	0.71 (0.59–0.86)
Southeast	1.22 (0.92–1.62)	1.00 (0.76–1.32)	0.80 (0.64–1.01)	0.72 (0.56–0.91)
South	1.94(1.42–2.65)	1.31 (0.95–1.81)	1.13 (0.88–1.45)	0.78 (0.60–1.02)
**Socioeconomic status**				
High	1	1	1	1
Middle	0.85 (0.65–1.12)	0.95 (0.71–1.29)	0.90 (0.72–1.12)	0.87 (0.70–1.09)
Low	1 (0.74–1.36)	1.05 (0.74–1.48)	0.96 (0.74–1.24)	0.90 (0.68–1.17)
**Education level**				
Elementary	4.07 (3.07–5.41)	5.84 (4.29–7.95)	1.89 (1.48–2.42)	2.30 (1.79–2.95)
Secondary	1.98 (1.52–2.59)	2.19 (1.63–2.93)	1.19 (0.95–1.48)	1.20 (0.95–1.50)
University	1	1	1	1
**Partner**				
With a partner		1		1
Without a partner		1.27 (1.03; 1.56)		1.03 (0.85–1.24)
**Sexual experience**				
Heterosexual		1		1
Same–sex		2.29 (1.78–2.96)		1.46 (1.15–1.86)
**Alcohol consumption (12 months)**				
No consumption		1		1
Consumption		5.65 (4.03–7.90)		3.83 (2.88–5.09)

*PR, Prevalence ratio. Model 1: Adjusted for sex, age, skin color, region, socioeconomic status, and education level. Model 2: Adjusted for sex, age, skin color, region, socioeconomic status, partner presence, education level, sexual experience, and alcohol use*.

## Discussion

We evaluated the smoking habits in a large, nationwide, young population in Brazil by employing a diverse sampling methodology and interviewing subjects outside their homes or schools to avoid response bias. We found that education level was lower in current daily smokers and higher in non-daily current smokers, regardless of social class or skin color/race. Some characteristics, such as not having a partner, alcohol use, and same-sex sexual experiences, were determined to be associated with an increased smoking prevalence.

We observed a higher prevalence of smoking (15.1%; 95% CI: 13.6–16.6) than was reported in a previous survey conducted in a similar age group in Brazil (5.7%; 95% CI: 5.3–6.2) (3, 34). As the studies were performed a few years apart, the differences in prevalence may reflect differences in the interview method rather than actual changes in the prevalence over time. Another factor that may explain the difference in prevalence rates may be the locations from which the young people were recruited, as the largest study enrolled adolescents from schools, while our study enrolled volunteers from the community. Hence, the difference could be due to response bias or to selection bias in previous studies that may have excluded participants with a higher prevalence of smoking, as they had already dropped out of school.

Young people with a low education level were up to 5 times more likely to report current smoking than those with a high education level. This finding corroborates other studies carried out in Brazil (24, 35) and worldwide (14, 36, 37). Education plays an important role in raising awareness about the damage and risks caused by tobacco; youth with more years of education might recognize the importance of not smoking (14). Youth who drop out of school early tend to begin assuming the responsibilities of adulthood earlier and are more vulnerable to tobacco addiction and other risks behaviors (38).

In this study, the group of participants who had a same-sex sexual experience had a higher rate of smoking than those who had not had such an experience. Similarly, sexual minorities exhibited higher frequencies of smoking than their heterosexual counterparts (39, 40). Although there is a decreasing tendency in the smoking prevalence among the general population, minority groups such as lesbians and gay men exhibit high and stable tendencies to smoke (41). Data collected from 112,537 adults in England also indicated that bisexual people are relatively more likely to smoke (42). A systematic review that analyzed the etiology of tobacco use in sexual minority populations revealed that internalized homophobia, reactions to the disclosure of one's sexual orientation, stress, depression, and alcohol use were associated with smoking (43). Accordingly, studies on smoking among the lesbian, gay, bisexual, and transgender (LGBT) population may help elucidate the specific risks and conditions to which this group is exposed and thus highlight the need for healthcare in this population (44, 45).

We found a strong association between alcohol consumption and tobacco smoking, with the prevalence of alcohol consumption being five times higher among current smokers than among non-smokers. While the association between alcohol consumption and cigarette smoking is well-known, it is important to emphasize that this association increases the risk of some cancers and cardiovascular diseases (15). In this context, alcohol is an important risk factor for the use of tobacco and other drugs, and young adults are vulnerable when in social situations in which the availability of alcohol can stimulate cigarette use (40, 46).

Our study supports previous results showing a higher prevalence of smoking in men despite the decreasing prevalence in both sexes (3, 9) and in individuals without a stable relationship (47). High prevalence of smoking in participants who did not have a partner could be associated with loneliness (47, 48).

Surprisingly, we observed no statistically significant differences among patterns of smoking according to socioeconomic status or skin color/race. The absence of associations concerning these two factors in our study could be due to the young age of the participants or to the fact that the majority of the included participants belonged to a lower socioeconomic level. Additionally, social class was measured according to the number of goods in the household rather than the family income, as previous studies have identified direct associations among smoking prevalence, education level, and income (socioeconomic class) (14, 49, 50). According to Bray et al., social inequalities promote vulnerability, which is determined by low education level, low income, and difficulty accessing healthcare, which lead to a higher rate of tobacco use (15%) compared to that in non-vulnerable populations (9%) (37). A meta-analysis that included 93 articles revealed that the prevalence of smoking was highest in low- and middle-income countries (1) and that smoking was associated with ethnicity in some studies (18, 35, 51), which conflicts with our findings.

The transition to adulthood is a period of intense change, as adolescents experience the end of high school and the beginning of University or employment. Smoking cigarettes constitutes a behavior that can start in adolescence and persist throughout life, leading to many social, physical, and economic consequences (52). Accordingly, global measures, including monitoring tobacco use; implementing prevention policies, such as public health interventions, individual and group counseling, behavioral support, and pharmacotherapy (53); protecting people from secondhand smoke; providing assistance and instruments to quit tobacco use; enforcing bans on tobacco advertising; promoting and sponsoring tobacco cessation campaigns; and raising taxes on cigarettes (4, 10, 54) have been deployed to promote cigarette smoking cessation. In Brazil, there are a large number of preventive campaigns focused on the risks of smoking and laws limiting the use of tobacco in public places and prohibiting access to and the consumption of tobacco products by those under the age of 18, although these restrictions are not respected or enforced by all merchants (13), as evidenced by a survey of 748 adolescents in Brazil, among whom 80.3% of those who smoked reported having easy access to cigarettes (20).

Our study has some limitations that must be considered. First, this multicenter study, including participants from all states of Brazil, was not population-based; instead, we used a convenience sampling method to select research units (primary healthcare units) in the state capitals in Brazil. To minimize selection bias, we invited participants from a variety of different settings, such as schools, primary healthcare units, the community at large, and a list of people living in the region who met the age criteria. Additionally, the study only included participants from the state capitals in Brazil. Although this could have introduced a bias in behavior, the included populations exhibited similar distributions of education, social class, and race/skin color that were representative of those recorded in the census of the Brazilian population. Although we had a large sample size, when we presented the prevalence stratified by sex or region, some estimates appeared to be unstable due to the small sample sizes in specific categories. This was a cross-sectional study, and causality could not be determined; we could only investigate the associations between the prevalence of smoking and the presence of different variables. Finally, we did not evaluate other tobacco-derived substances, such as e-cigarettes, cigarillos, filtered cigars, pipe tobacco, snus, and other smokeless tobacco products.

In conclusion, although the smoking prevalence has been decreasing in the Brazilian population, we demonstrated that younger people have a higher prevalence of current and past smoking. We also found that smoking is associated with education level regardless of socioeconomic status. Some specific factors, such as same-sex sexual experiences, male sex, and alcohol use, are associated with an elevated prevalence of smoking. The damage caused by smoking is undeniable, and even with all of the campaigns and policies focused on reducing the use of this substance, smoking remains a problem among the Brazilian youth, especially among people with same-sex sexual experiences. Campaigns targeting specific populations are worthwhile, and studies that address this specific behavior are needed to understand the behaviors associated with smoking in specific sexual minority groups.

## Data Availability Statement

The raw data supporting the conclusions of this article will be made available by the authors, without undue reservation.

## Ethics Statement

The studies involving human participants were reviewed and approved by Research Ethics Committee of the Moinhos de Vento Hospital (number 1607032). Written informed consent from the participants' legal guardian/next of kin was not required to participate in this study in accordance with the national legislation and the institutional requirements.

## Author Contributions

EB and EW contributed substantially to the conception and design of the work. TD, FM, and GP contributed to the critical analysis. EB wrote the first draft. MB conducted the data analyses. All authors contributed to the article and approved the submitted version.

## Funding

This work was supported by the Hospital Moinhos de Vento through the Institutional Development Support Program of the Brazilian National Health System (PROADI-SUS) under the Ministry of Health of Brazil.

## Conflict of Interest

The authors declare that the research was conducted in the absence of any commercial or financial relationships that could be construed as a potential conflict of interest.

## Publisher's Note

All claims expressed in this article are solely those of the authors and do not necessarily represent those of their affiliated organizations, or those of the publisher, the editors and the reviewers. Any product that may be evaluated in this article, or claim that may be made by its manufacturer, is not guaranteed or endorsed by the publisher.
